# Immunoregulation effects of different γδT cells and toll-like receptor signaling pathways in neonatal necrotizing enterocolitis

**DOI:** 10.1097/MD.0000000000006077

**Published:** 2017-02-24

**Authors:** Lei Hui, Yi Dai, Zhi Guo, Jiahui Zhang, Fang Zheng, Xiangli Bian, Zhimin Wu, Qin Jiang, Miaomiao Guo, Ke Ma, Jinping Zhang

**Affiliations:** a Department of Pediatrics, Shanghai 6th People's East Hospital, Jiao Tong University, Pudong Nanhui New City; b Department of Neonatology, Children's Hospital of Fudan University; c Emergency Department, Shanghai 6th People's East Hospital, Jiao Tong University, Pudong Nanhui New City, Shanghai, China.

**Keywords:** gamma delta T cells, intraepithelial lymphocytes, neonatal necrotizing enterocolitis, preterm infants, TLR4, TLR9

## Abstract

The aim of the study was to observe cytokine and T-cell-related toll-like-receptor (TLR) changes in intestinal samples of neonatal necrotizing enterocolitis patients.

Four necrotic bowels were collected from neonatal NEC patients with gestational ages of 28 to 29 weeks in our hospital, whereas 4 neonatal patients who underwent intestinal atresia surgery served as the controls. Intestinal flora was examined and IL-1, IL-2, IL-4, IL-6, IL-8, IL-10, TNF-α, IFN-γ, and IL-17 expressions in resected intestine samples, as well as in isolated *gamma delta T (γδT) cells,* were analyzed immunohistochemically and via quantitative RT-PCR. *γδT cells were isolated from the intestinal intraepithelial lymphocytes (IELs) and their* TLR4/TLR9 distribution in the intestinal tissues was determined by flow cytometry.

The bacterial flora of the neonatal NEC patients’ contained significantly higher amounts of Gram-negative *Enterobacteriaceae*, *Klebsiella,* and *Bacteroides* but anaerobic Gram-positive *Bifidobacteria* occurred significantly less in the NEC than the control group. IL-1, IL-2, IL-4, IL-6, IL-8, IL-10, TNF-α, IFN-γ, and IL-17 expressions in the resected intestine samples and in isolated *γδT cells* were enhanced in NEC samples compared to the controls. *γδT cells were less prevalent in NEC-derived intestinal tissues, but their* TLR4/TLR9 expressions were significantly enhanced.

The changed bacterial flora in preterm neonatal NEC patients led to an obvious inflammation of the intestines, which was accompanied by reductions of *γδT cell localizations to the intestine and a shift of their surface expressions to TLR4 and TLR9.*

## 1 Introduction

Neonatal necrotizing enterocolitis (NEC) is an intestinal disease with high morbidity (2.6% to 28%), mortality (10% to 50%), and late disability rates. NEC accounted for 1.0% to 7.7% of neonatal intensive care unit (NICU) admissions, 90% occurring in preterm births.^[1]^ Previous studies suggested NEC preterm infants exhibit excessive inflammatory responses to intestinal microbials, which alters the protective barrier in the intestine^[2]^ and may lead to sepsis, which depending on its severity occurs in 40% to 60% of preterm NEC cases.^[3]^*Gamma delta T cells (γδ T cells) are T cells with distinctive T-cell receptors (TCRs) on their surface. Most T cells are the alpha beta (αβ) type consisting of 2 glycoprotein chains (α and β) in their TCR. Gamma delta (γδ) T cells have a TCR consisting of 1 gamma (γ) and 1 delta (δ) chain.* Depending on their TCR variable regions, *γδ T cells are further divided into Vγ9Vδ2 (Vδ2 cells) and* V*δ*1 combined with various V*γ* elements *subclasses (*V*δ*1 cells)*, which differ in their ligands. γδT cells have been reported to express/co-express also a wide range of TLRs.*^[4]^ γδT cells account for only 1% to 2% of the total T cells in the peripheral circulation, mainly consisting of Vδ2 T cells, *but comprise a majority of the lymphocyte population in the gut and in other epithelial mucosa, and are termed intraepithelial lymphocytes (IELs).*^[5,6]^ After antigen stimulation, γδT cells release IFN-γ and TNF-α, activate a congenital immune response, and also coordinate an acquired immune response mediated by αβT cells; γδT cells also have immuno protective effects on the intestinal parasitic infection.^[7]^

*It has been shown that γδT cells in contrast to αβT-cells are precociously active in human neonates and are the* first T cells to develop.^[8]^ However, the excessive response of preterm infants to intestinal microbials has been attributed to enhanced activity of the TLR4,^[9]^ since this bacterial lipopolysaccharide (LPS) recognizing factor's expression is enhanced in preterm infants.^[2,10]^ It is noteworthy that different TLRs were reported to play different roles in the immune mechanisms of NEC, since TLR4 gene knockout could prevent NEC in mice,^[11]^ whereas TLR9 was shown to be an NEC reducing factor.^[12,13]^

In the present study, we focused on NEC-related TLR4 and TLR9 expression of *γδT cells in* preterm birth NEC patients.

## 2 Patients and methods

The ethical committee of the 6th Hospital of the Jiaotong University approved the study. The parents of all patients gave written informed consent, and our study was carried out in accordance with the approved guidelines. Four necrotic bowels were collected from neonatal NEC patients with gestational ages of 28 to 29 weeks, and 4 neonatal patients who underwent intestinal atresia surgery served as the controls.

### 2.1 Intestinal specimen collection and pretreatment

Necrotic small/large intestinal tissues were excised during the operation and placed in tissue culture solution. After removal of mesenteric fat and mucus, the intestines were cut into 5 mm segments and washed 3 times with lymph extraction fluid after which the lymph extraction fluid was stir cultured for 20 minutes at 37°C and then filtered. The cells were purified with 40% isotonic cell separation fluid and again cultured for 30 minutes. After centrifugation at 1000 rpm for 10 minutes, the cell supernatant fluid was lymphocyte rich.

### 2.2 Distribution of main intestinal bacterial strains in NEC and control groups

None of the mothers had experienced bacterial vaginosis during pregnancy. The placenta samples were collected using sterile centrifuge tubes immediately after delivery. To avoid the placenta being contaminated by the vagina, samples were collected from the inner surface of the placenta. The first feces of the neonates were also collected in the delivery room using sterile cotton swabs. Sterile cotton swabs in the same environment were also analyzed as negative controls. All samples were immediately stored at –20°C. Total DNA was extracted from 1 g of placenta or feces, or 1 cotton swab as previously described,^[14]^ with minor modifications. Briefly, samples were simultaneously treated with lysozyme (1 mg/mL) and lyticase (0.16 mg/mL). Subsequently, samples were treated with sodium dodecyl sulfate (1%) and cetrimonium bromide (1%). Three liquid nitrogen freeze/thaw cycles were also performed to ensure homogeneity of the lysed cell samples. The concentration of extracted DNA was determined using a spectrophotometer (NanoDrop ND 1000, Thermo Fisher).

In all polymerase chain reaction (PCR) amplifications, reactions were performed with rTaq MasterMix (TaKaRa, China), in a total volume of 50 μL using approximately 50 ng DNA template. A modified primer set (338F and 907R) was used according to a metagenomics database.^[15]^ The resulting 16S rRNA sequences were analyzed with the Silva v108 database using the mothur program v.1.25.1 (www.mothur.org/wiki/Main_Page). The operational taxonomic units (OTUs) of the 16S rRNA gene sequences were determined using a 3% cut-off. To determine the phylogenetic position of the 16S rRNA genes, sequences were compared with available database sequences via a BLAST search; related sequences were obtained from the National Center for Biotechnology Information nonredundant database. The taxonomic information was further confirmed by the online analysis tool EzTaxon.^[16]^

### 2.3 Purification of human intestinal γδT cells

Intestinal γδT cells were isolated with Percoll density gradients according to the method described in a previous publication.^[17]^ After the initial isolation, specific intestinal lymphocyte subgroup antibodies were used for further separation of TCR (GL3) and TCRβ (H57) cells. The lymphocytes were sealed off for 15 minutes at 4°C using biotin labeled anti-γδT cell antibodies and cleaned 3 times with PBS through biotin magnetic beads.

### 2.4 Detection of intestinal γδT cell subgroups and TLR distribution with flow cytometry

Purified human or mice intestinal γδT cells were incubated with fluorescent labeled anti-TCR-Vδ1, anti-TCR-Vδ2 (PE labeling), and TLR4/9 antibodies (APC labeling) and further separated by flow cytometry with a FACS Aria II flow cytometer (BD Biosciences).

### 2.5 Expression analyses of cytokines in intestinal epithelial cells by immunohistochemistry

Some of the surgically excised large and small intestine samples were stored in a cutting fluid and frozen in liquid nitrogen. The frozen tissues were then cut into 6 μm slices, which were placed in propanol at 4° for 15 minutes and then incubated in 3% H_2_O_2_ for 15 minutes at room temperature to eliminate endogenous peroxidase activity. After washing 3 times with PBS, the sections were blocked with normal goat serum for 30 minutes at 37°C and incubated with primary antibodies against IL-1, IL-2, IL-4, IL-6, IL-8, IL-10, IL-17, TNF-α, and IFN-γ for 1 hour at 37°C. After washing the slides 3 times with PBS, the sections were incubated with diluted biotin labeled secondary antibody after which appropriately diluted streptavidin labeled horseradish peroxidase was added for 30 minutes. After washing with PBS again, the DAB chromogenic reagent (Wuhan Boster Biological Co., Ltd.) was added and the nuclei were stained with hematoxylin.

### 2.6 RT-PCR analysis of intestinal inflammatory factor transcriptions

The purified intestinal γδT cells were dissolved in pyrolysis liquid, and RNA was extracted using an RNeasy mini kit in strict accordance with the manufacturers’ instructions (Qiagen). cDNA was constructed using a reverse transcription kit (Roche). IL-1, IL-2, IL-4, IL-6, IL-8, IL-10, IL-17, TNF-α, and IFN-γ primer were designed, and suitable PCR conditions were determined in the pre-experiments. Finally, semiquantitative real-time PCR was performed using the Fast Start Universal SYBR Green Master reagent (Rox) (ABI) with ß-actin serving as the internal control.

### 2.7 Statistical analysis

The statistical analysis was performed using SPSS for Windows (Version 13.0. Chicago, SPSS Inc.). All data are presented as the mean ± SD. For differences, a Student-*t* test was employed, with the level of statistical significance set at *P* < 0.05.

## 3 Results

### 3.1 Bacterial species in the NEC and control groups

**Figure 1:**
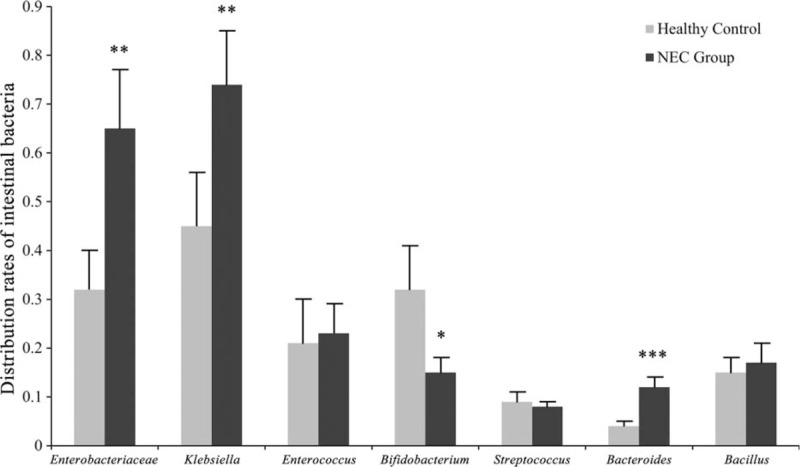
Rates of indicated bacterial species in NEC and control intestines. NEC = neonatal necrotizing enterocolitis.

The bacterial compositions in NEC and the controls showed different patterns. The proportion of Gram-negative *Enterobacteriaceae*, *Klebsiella,* and *Bacteroides* were higher, whereas the proportion of anaerobic Gram-positive *Bifidobacteria* was lower in the NEC than in the control group (Fig. 1).

### 3.2 Immunohistochemical analyses of inflammatory cytokines in human NEC and control-derived intestinal tissues

**Figure 2:**
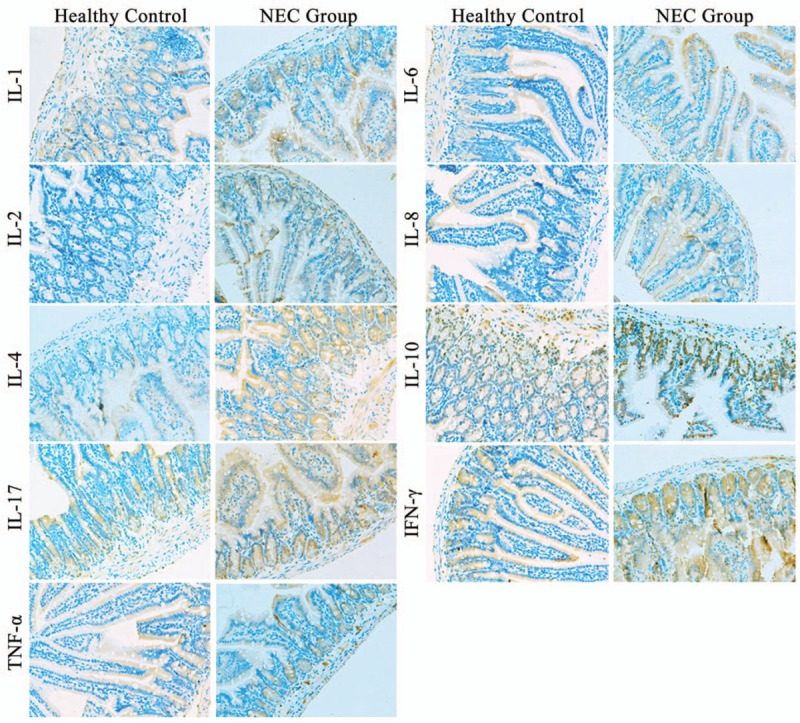
Immunohistochemical analysis of inflammatory factors in human NEC and control-derived intestinal tissues. NEC = neonatal necrotizing enterocolitis.

Immunohistochemical results showed that the expression of the inflammatory factors IL-1, 2, 4, 6, 8, 10, 17, IFN-γ, and TNF-α in necrotic intestinal tissue of NEC neonatal patients was more pronounced than in the control group (Fig. 2).

### 3.3 Increased intestinal endothelium inflammatory factor activities in the human NEC group

**Figure 3:**
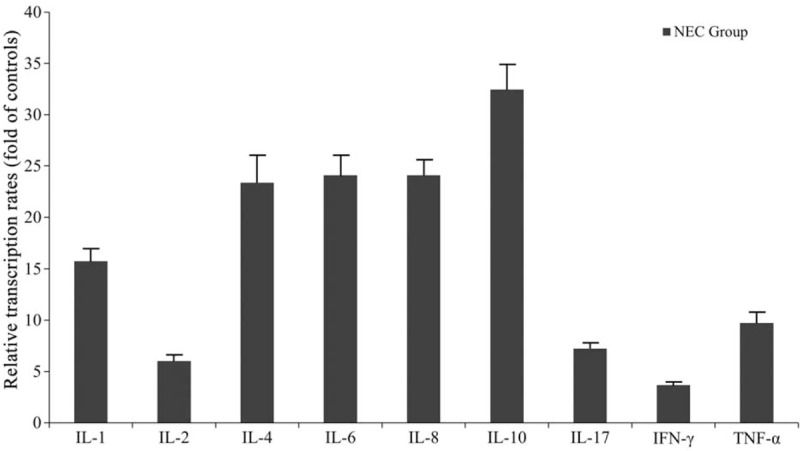
Transcription rates of cytokines in NEC intestine-derived γδT cells. Data are shown as the fold of the control. NEC = neonatal necrotizing enterocolitis.

In order to analyze further the expression of inflammatory cytokines in NEC-derived intestine samples, we determined the transcription rates of IL-1, IL-2, IL-4, IL-6, IL-8, IL-10, IL-17, IFN-γ, and TNF-α in purified γδT cells. As shown in Fig. 3, all factors were transcribed at significantly higher levels in γδT cells isolated from human NEC intestinal tissues compared to the control group.

### 3.4 Distribution of γδT and TLR cell subclasses in human NEC tissues

**Figure 4:**
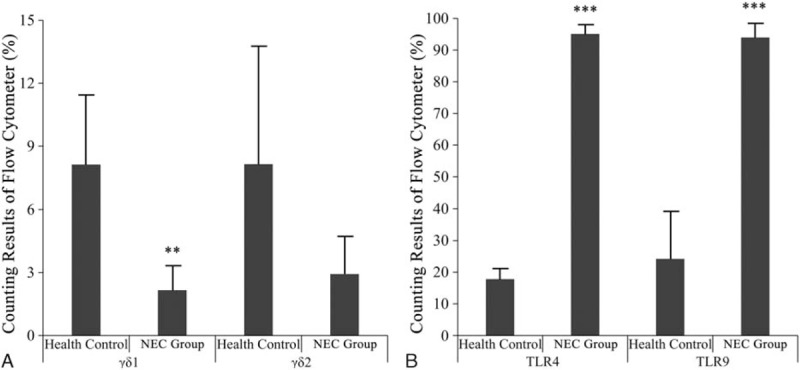
Proportions of γδ1 and γδ2 γδT cells, as well as TLR4, TLR9 expressing γδT subclass cells, derived from human NEC and control intestinal tissues. NEC = neonatal necrotizing enterocolitis, TLR = T-cell related toll-like-receptor.

The γδ1 and γδ2 cell counts were reduced but the proportions of TLR4 and TLR9 expressing γδT cells were significantly enhanced in intestinal tissues derived γδT cells of NEC compared to the controls, indicating that in the intestinal γδT cell population of the NEC group, although diminished, a shift to more TLR4 and TLR9 receptor expression took place (Fig. 4).

## 4 Discussion

Our results revealed that the proportions of Gram-negative *Enterobacteriaceae*, *Klebsiella,* and *Bacteroides* were enhanced, whereas the proportion of anaerobic Gram-positive *Bifidobacteria* was diminished in NEC compared to the control group (Fig. 1), which is in agreement with previous reports that the microbial flora in the intestine of premature infants often consists of pathogenic species, including *Klebsiella* and *Enterobacteriaceae*, with a reduced colonization by normal commensals such as *Bifidobacterium* and *Lactobacillus* species.^[18,19]^ This inappropriate colonization of the intestine in preterm infants has been attributed to be the cause of NEC^[20]^ and TLR4, which is the receptor for bacterial endotoxins and bacterial long chain lipids, is thought to play an essential role in the pathogenesis of NEC.^[9,11]^ It has been proposed that the inability of the intestine to downregulate exaggerated TLR4 signaling and become tolerant to the luminal bacteria induces intestinal injury by apoptosis and reduced healing due to impaired enterocyte proliferation and migration in NEC patients.^[21]^

Our findings are in agreement with these findings, since TLR4 was significantly upregulated in γδT cells of the preterm IELs, but also TLR9 expression was enhanced to a similar degree, *which is contrary to a previous study in which TLR4 and TLR9 were reciprocally expressed in an NEC mouse model.*^[13]^*The findings indicate that in NEC patients TLR4 expression was partly reduced by upcoming TLR9 activity since* TLR9, which after activation, downregulates TLR4 activity was shown to be an NEC ameliorating factor.^[12,13]^ In our study, expression of the pro-inflammatory/inflammatory/antiinflammatory cytokines IL-1, IL-2, IL-4, IL-6, IL-8, IL-10, IL-17 as well as IFN-γ and TNF-α were significantly upregulated in NEC-derived intestine γδT cells and tissue samples compared to the controls, which is in agreement with previous studies.^[22,23]^ The highest upregulation was detectable for IL-10, which is an anti-inflammatory cytokine, indicating that also protective mechanisms take place during NEC development in NEC patients.^[24]^

## 5 Conclusions

In the intestines of our preterm NEC infants, the proportion of Gram-negative *Enterobacteriaceae*, *Klebsiella,* and *Bacteroides* were higher, whereas the proportion of anaerobic Gram-positive *Bifidobacteria* was lower than that in the control group, which was accompanied by elevated cytokine expression in the resected intestine samples and γδT cells. γδ1 and γδ2 cell localizations in the IELs of the resected intestines were significantly reduced, but their surface receptor expressions shifted to TLR4 and TLR9.
